# The residual STL volume as a metric to evaluate accuracy and reproducibility of anatomic models for 3D printing: application in the validation of 3D-printable models of maxillofacial bone from reduced radiation dose CT images

**DOI:** 10.1186/s41205-015-0003-3

**Published:** 2015-11-27

**Authors:** Tianrun Cai, Frank J. Rybicki, Andreas A. Giannopoulos, Kurt Schultz, Kanako K. Kumamaru, Peter Liacouras, Shadpour Demehri, Kirstin M. Shu Small, Dimitris Mitsouras

**Affiliations:** 1 Department of Radiology, Brigham and Women’s Hospital, Boston, MA USA; 2 The Ottawa Hospital Research Institute and Medical Imaging, Ottawa, ON Canada; 3 Department of Radiology, University of Ottawa, Ottawa, ON Canada; 4 Toshiba Medical Research Institute USA, Vernon Hills, IL USA; 5 Department of Radiology, Juntendo University, Tokyo, Japan; 6 Department of Radiology, Walter Reed National Military Medical Center, Bethesda, MD USA; 7 Division of Musculoskeletal Radiology, Russell H. Morgan Department of Radiology and Radiological Sciences, Johns Hopkins University, School of Medicine, Baltimore, MD USA; 8 Applied Imaging Science Lab, Department of Radiology, Brigham and Women’s Hospital, Boston, MA USA

## Abstract

**Background:**

The effects of reduced radiation dose CT for the generation of maxillofacial bone STL models for 3D printing is currently unknown. Images of two full-face transplantation patients scanned with non-contrast 320-detector row CT were reconstructed at fractions of the acquisition radiation dose using noise simulation software and both filtered back-projection (FBP) and Adaptive Iterative Dose Reduction 3D (AIDR3D). The maxillofacial bone STL model segmented with thresholding from AIDR3D images at 100 % dose was considered the reference. For all other dose/reconstruction method combinations, a “residual STL volume” was calculated as the topologic subtraction of the STL model derived from that dataset from the reference and correlated to radiation dose.

**Results:**

The residual volume decreased with increasing radiation dose and was lower for AIDR3D compared to FBP reconstructions at all doses. As a fraction of the reference STL volume, the residual volume decreased from 2.9 % (20 % dose) to 1.4 % (50 % dose) in patient 1, and from 4.1 % to 1.9 %, respectively in patient 2 for AIDR3D reconstructions. For FBP reconstructions it decreased from 3.3 % (20 % dose) to 1.0 % (100 % dose) in patient 1, and from 5.5 % to 1.6 %, respectively in patient 2. Its morphology resembled a thin shell on the osseous surface with average thickness <0.1 mm.

**Conclusion:**

The residual volume, a topological difference metric of STL models of tissue depicted in DICOM images supports that reduction of CT dose by up to 80 % of the clinical acquisition in conjunction with iterative reconstruction yields maxillofacial bone models accurate for 3D printing.

## Background

Medical 3D printing is currently undergoing a rapid transition from niche applications to more routine utilization, particularly in reconstructive surgeries as well as cardiovascular and neuro-interventions [1]. This increased utilization is secondary to lower costs and greater awareness that 3D printing can enhance patient care [2–4]. Translating a new technology from the research to clinical domain however first requires standardization and validation.

A key validation component involves metrics amenable for universal application of accuracy and reproducibility testing across equipment platforms and acquisition protocols. One example is that of quality assurance phantoms used to establish the accuracy of images generated by an imaging system based on standardized measurements, such as the American College of Radiology Computed Tomography phantom [5]. Similarly, for 3D printing, one strategy may include carefully developed phantoms that, once printed, can undergo standardized tests and measurements to establish the accuracy of the 3D printing hardware and software pipeline. This strategy does not readily translate to the relative quality assessment measures needed to assess individual clinical scans obtained with varying clinical protocols and that depict different tissues and pathologies.

The signal-to-noise ratio (SNR) and contrast-to-noise ratio (CNR) are metrics of the diagnostic capacity of an individual scan used to this end. They take into account both the acquisition protocol (e.g., echo and repetition time in MRI; tube current and potential in CT), as well as the underlying tissue properties (e.g., T_1_ and T_2_ properties; X-ray attenuation). These metrics strongly contribute to the determination of an individual study’s diagnostic accuracy. The present study is based on the author’s experience that a similar approach is necessary to assess the expected accuracy of 3D printed models generated from individual clinical data sets. This was underscored by a recent study that reported a >1 mm variation in anatomical properties of a skull standard tessellation language (STL) model generated by three independent, specialist institutes from a single DICOM CT dataset [6].

3D printing of bone from CT images currently accounts for the majority of 3D printing applications in medicine, starting with maxillofacial and neurosurgical applications [6, 7]. We consider 3D printed models as the best method to select locations of appropriate and optimal osteosynthesis for full-face transplantation [8]. 3D models are also used to reduce surgical time after trauma and improve outcomes in patients with complex bone and joint injuries such as acetabulum and posterior wall pelvic fractures [9–11], and to test the effectiveness of novel surgical tools for total shoulder arthroplasty [12]. For pelvic bone tumors, 3D printing has been used to generate patient-specific bone cutting instrumentation to enhance accuracy and potentially improve resection [13]. In spine intervention, 3D printed models of bone have assisted in the management of structural, traumatic and neoplastic diseases by helping to confirm pedicle screw placement [14–18], and in congenital and acquired pediatric orthopedic disorders they have aided in better anatomical understanding of the lesion [19, 20]. Going forward, clinical applications of 3D-printed models will require a confidence metric that assesses the relationship between the imaging parameters used to acquire the source DICOM images and the resulting STL model derived from them.

The purpose of this work was to develop such a metric, namely the “residual STL volume” and use its topology for the assessment of maxillofacial bone models derived from CT images at multiple simulated radiation doses. CT data was acquired using a standard clinical protocol and simulated noise was added in the raw data space (sinogram) using a previously validated approach [21]. The degradation of image quality among those images with simulated lower radiation dose was carefully assessed to determine the impact on the novel 3D printing accuracy metric.

## Methods

### Patients

Two full-face transplantation patients enrolled in clinical trial NCT01281267 were retrospectively evaluated in this study. Both patients signed the written informed consent approved by the Institutional Human Research Committee. Briefly, patient 1 was a 30-year-old male who was involved in a motor vehicle accident that resulted in a high voltage electrical injury to his face. The patient underwent full-face transplantation after multiple conventional reconstructive surgeries. Patient 2 was a 25-year-old male who had catastrophic loss of facial tissues after high voltage injury. Other surgical options were exhausted after 20 procedures including multiple flaps covered with skin grafts, and the patient underwent full-face transplantation. These patients have been previously reported [8, 21], and images using the acquisition and noise simulation described below have been used to optimize CT protocols for surgical planning [21].

### CT acquisition and noise simulation

Both patients were imaged with a first generation 320 × 0.5 mm detector row CT (Aquilion ONE, Toshiba Medical Systems Corporation, Tochigi-ken, Japan). The baseline study for each patient was without intravenous contrast and was acquired at 80 kV and 155 mA with a 500 millisecond gantry rotation time. Sinogram data were archived as part of the trial protocol, and simulated sinogram data at lower mA were generated using previously described [21] and validated [22] manufacturer provided noise simulation software. For each patient, 5 sinograms were generated: that of the baseline clinical acquisition (155 mA) without any modification (referred to as “100 % dose” below), plus four additional sinograms at 50, 40, 30 and 20 % of the radiation dose of the baseline acquisition.

### CT image reconstruction

Two image reconstruction algorithms, filtered back projection (FBP) and the manufacturer’s Adaptive Iterative Dose Reduction 3D (AIDR3D) [21] were used to reconstruct images from each of the 5 sinograms for each patients. Thus, for each patient, 10 image data sets were produced, 5 reconstructed with FBP and 5 with AIDR3D. The recommended manufacturer FBP kernel for soft tissue display (FC41) was used. All images were reconstructed at 0.5 mm slice thickness and 0.5 mm increment. For succinctness, below we refer to a “configuration” as the image dataset resulting from applying one reconstruction algorithm to one sinogram.

### Signal-to-noise ratio assessment

**Fig. 1:**
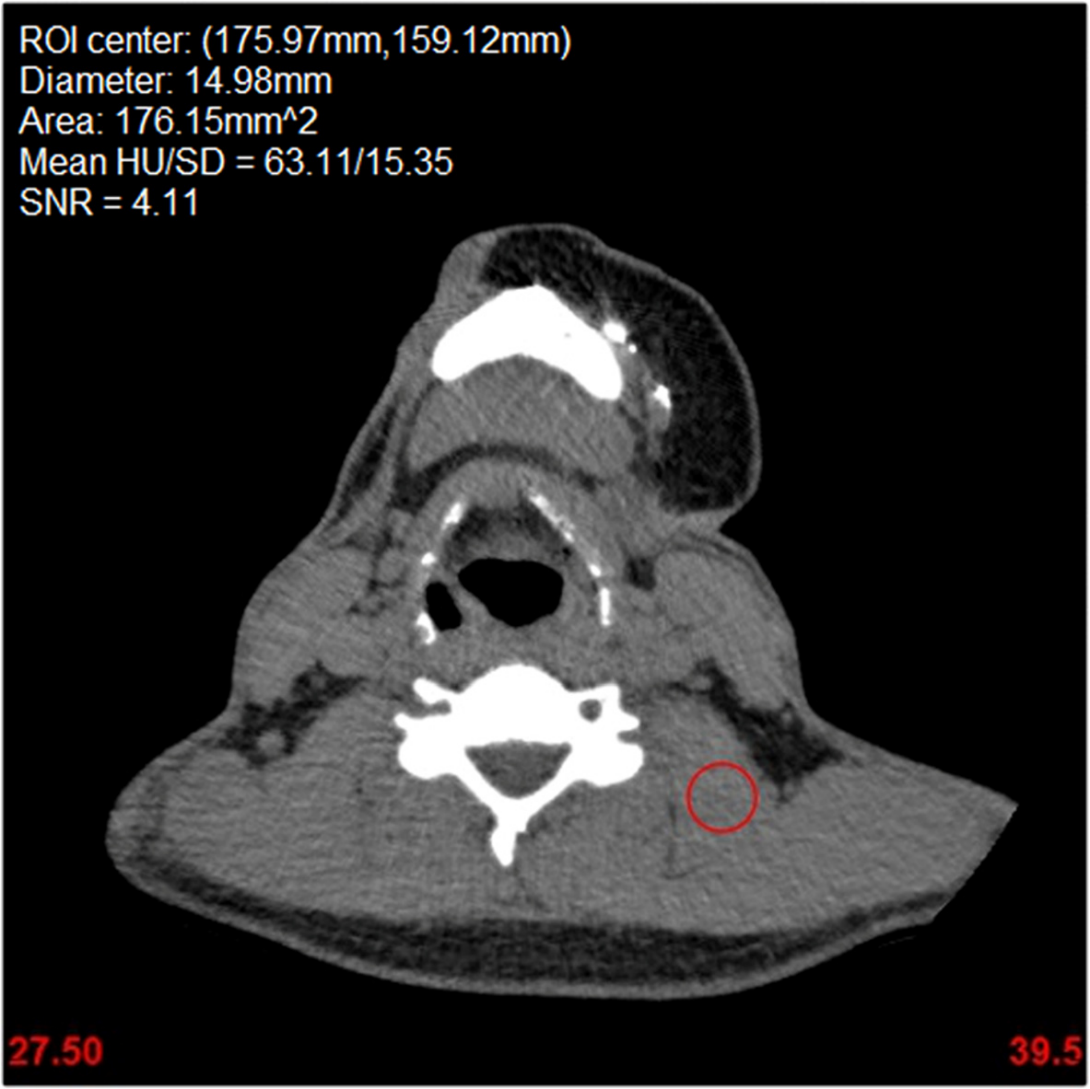
Single axial slice of the clinical acquisition (100 % radiation dose) reconstructed with AIDR3D for one patient showing the disk-shaped ROI (7.49 mm *radius circle* shown in *red*) used for SNR measurement in the levator scapulae muscle. The ROI was identically replicated using software to the remaining 9 radiation dose/reconstruction algorithm configurations

Signal-to-noise for each configuration was calculated from the mean and standard deviation (SD) of Hounsfield Units (HU) in disk-shaped regions-of-interest (ROI) placed in the semispinalis capitis, masseter, sternomastoid and trapezius muscles. The diameter of each ROI was approximately 7.5 mm (Fig. 1). Signal-to-noise ratio (SNR) was calculated by dividing the mean HU of each ROI by its SD. Identical ROI measurements were made across all 10 configurations for each patient to compare attenuation and SNR differences between simulated doses and reconstruction methods. For this purpose a software program was developed to allow selection of the ROI center and radius in one configuration, and then apply it to the identical location in each of the other 9 configurations. The initial manual placement of the ROI for each muscle was performed after review of all 10 datasets, with care taken to avoid artifacts or other tissues.

### Generation of STL models

All 20 image datasets were imported in commercial medical 3D printing software (Mimics, Materialise NV, Belgium) using the “loseless” compression setting. Maxillofacial bone segmentation was performed identically across all datasets using thresholding with lower attenuation = 226 HU and upper attenuation = 3071 HU [23, 24]. Data was subsequently cropped to a region similar to the allograft of a full facial transplantation [25], with craniocaudal extent from the superior border of the nasal bone to the inferior margin of the mandible and anterior to posterior extent including the apex nasi to the superior border of the mandible. Cropping was automatically applied identically to all 10 configurations for each patient also employing an automated software program. These steps resulted in a voxel “mask” that demarcated all voxels included in the segmentation within the cropped volume. A final step was used to select only those demarcated voxels that were connected; this was achieved using the software “region growing” tool with the default 6-voxel connectivity. Finally, the automated STL generation method supplied by the software was used at the default “optimal” quality to generate the surface enclosing the segmented voxels.

### The residual volume

**Fig. 2:**
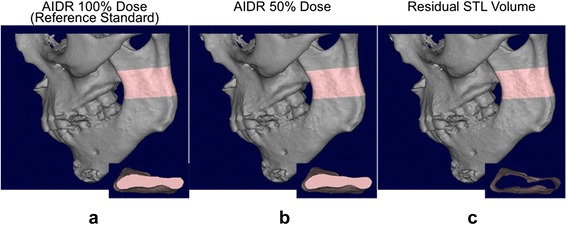
STLs derived from CT images of one patient reconstructed at different mAs (**a**, **b**, 100 and 50 % radiation dose, respectively, using the AIDR3D reconstruction algorithm). A portion of the mandible is *highlighted in pink* and the volume enclosed by the STL for that portion is shown in the *inset* from a superior-to-inferior view. Topologic subtraction of the 50 % dose volume is performed to yield the residual volume, i.e., the physical volume occupied by either the 100 or 50 % dose STL but not the other (**c**)

The STL model derived from the original tube current setting (155 mA, 100 % dose) and reconstructed with the AIDR3D algorithm for each patient, referred to as the reference configuration, was considered the reference bone STL. For all other configurations, a “residual STL volume”, denoted *STL*_*R*_, was calculated as the volume of physical space occupied by either the STL derived by the reference or the alternative configuration dataset, but not both. Mathematically, this is expressed as the subtraction of the intersection of the two volumes from their union:

$$ ST{L}_R=\left(ST{L}_{ref}{\displaystyle {\displaystyle \cup ST{L}_{alt}}}\right)-\left(ST{L}_{ref}{\displaystyle \cap ST{L}_{alt}}\right), $$

where *STL*_*ref*_ is the volume enclosed by the STL derived from the reference configuration, and *STL*_*alt*_ is the volume enclosed by the STL derived from the alternative configuration (Fig. 2). In addition to the absolute volume in units of cm^3^, two additional properties of the residual volume where used to characterize its overall topology. The first was its volume as a relative fraction of the *STL*_*ref*_ volume. For the second, we calculated an average thickness for the residual volume as the ratio of volume to surface area. This latter metric can be interpreted as a guide of the topology of the difference in the STLs derived from the different configurations; if the difference involves a large “lump” of bone not correctly segmented, for example missing due to reduced HU in a particular region, then the volume will be large, but the surface area enclosing it will be relatively small. Vice versa, if the difference is a slightly thicker or thinner segmentation, then the volume will be small and the surface area will be large, like the surface area of a thin foil.

### Statistical analysis

Statistical analyses were performed in RStudio version 0.98.945 (RStudio, Inc, Boston, MA, USA). The HU and SNR of each configuration was summarized by the mean and SD. One-way ANOVA with post-hoc multiple comparisons was used to compare image SNR and residual volume characteristics between different reconstructions algorithms at each tube current plus at different tube currents for each reconstruction algorithm. To examine whether AIDR3D has a higher SNR than FBP across different dose levels, linear regression models were used, with the paired average difference between AIDR3D and FBP being the response, and dose being the independent variable.

## Results

### Signal-to-noise ratio assessment

**Fig. 3:**
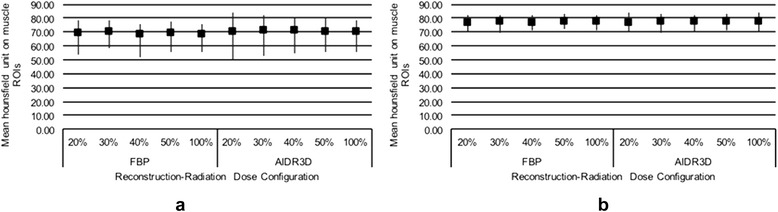
Mean HU of all muscle ROIs for each configuration in the first (**a**) and second (**b**) patient

**Fig. 4:**
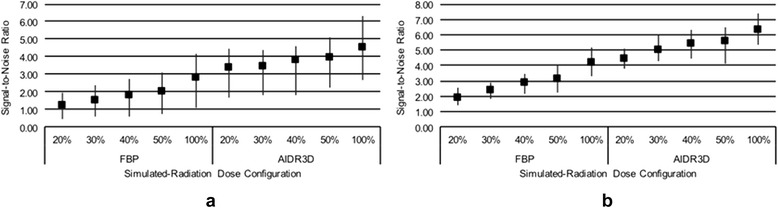
Image SNR for each reconstruction/radiation dose configuration in the first (**a**) and second (**b**) patient included in this study. SNR is calculated as the mean divided by the SD of HUs in ROIs placed in each of four facial muscles, averaged over all muscles

Mean attenuation for all muscle ROIs in the two patients were 68.6 ± 6.2 to 70.8 ± 6.6 across different configurations and did not significantly differ among them (Fig. 3). SNR decreased for both AIDR3D and FBP as the simulated noise was increased. The SNR of AIDR3D was significantly higher than that of FBP at all configurations, although the improvement decreased slightly as dose increased, at an average of 0.048 reduction in SNR difference per 10 % increase in dose. At 100 % dose, the average SNR difference between AIDR3D and FBP was 1.71 (*p* <0.0001; Fig. 4). Furthermore, AIDR3D-reconstructed images from the 20 % simulated dose data had a higher SNR (SNR = 3.38 ± 0.64 and 4.42 ± 0.36 in patients 1 and 2, respectively) than that using FBP on the 100 % dose data (SNR = 2.83 ± 0.90 and 4.21 ± 0.54 in patients 1 and 2, respectively).

### Bone and residual volume STL characteristics

**Fig. 5:**
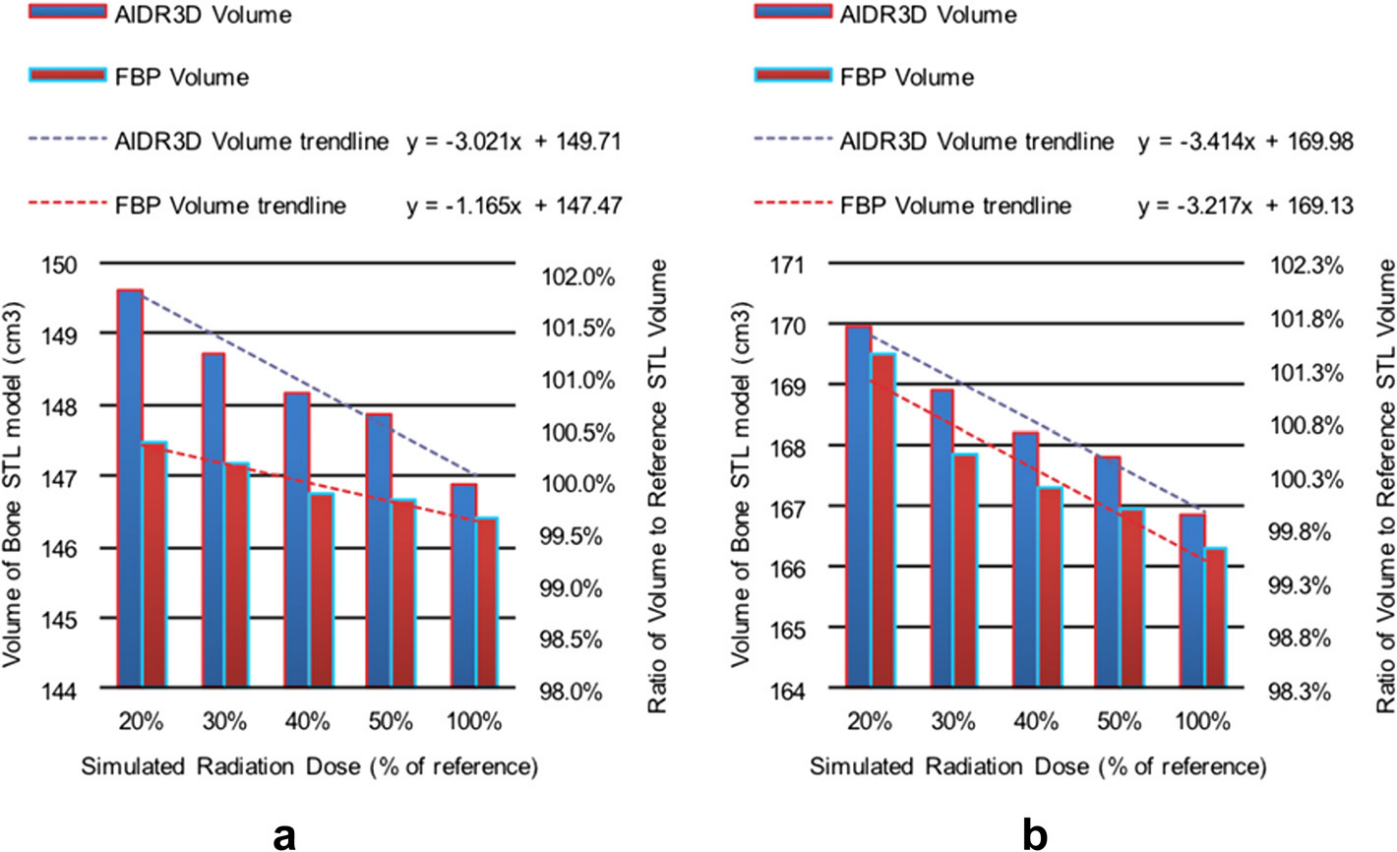
Volume of bone STL model for each reconstruction/radiation dose configuration in the first (**a**) and second (**b**) patient included in this study shown both as an absolute measurement (*left y-axis*) as well as with respect to the volume of the reference bone STL model derived from AIDR3D reconstruction of the clinically-acquired data (100 % radiation dose)

An STL model of the bone was successfully created from all 20 datasets. As image noise increased (lower dose), so did the STL volume by roughly 0.3 cm^3^ for AIDR3D and 0.12 cm^3^ for FBP reconstructions per 10 % noise increment in patient 1 (*p =* 0.02 and 0.06), and 0.34 cm^3^ and 0.32 cm^3^ for patient 2 (*p =* 0.03 and 0.08; Fig. 5). AIDR3D configurations resulted in overall slightly higher STL volumes than FBP configurations at the corresponding mAs, on average 0.92 and 0.45 % larger for patients 1 and 2 respectively.

**Fig. 6:**
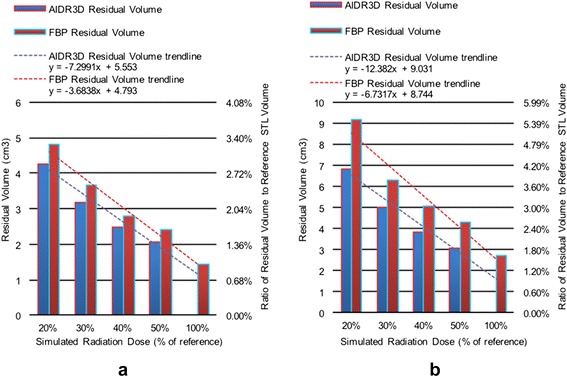
Residual volumes resulting from topological subtraction of the STL model derived for each reconstruction/radiation dose configuration from that derived for the reference standard configuration (AIDR3D reconstruction, 100 % dose) in the first (**a**) and second (**b**) patient included in this study shown both as an absolute measurement (*left y-axis*) as well as with respect to the volume of the reference bone STL model

The residual volume resulting from topological subtraction of the alternative configuration bone STL models from the reference model (AIDR3D at 100 % dose configuration) decreased with increasing radiation dose regardless of reconstruction algorithm and was slightly lower for AIDR3D compared to FBP configurations at all simulated radiation dose levels (Fig. 6). The residual volume decreased at a rate of 0.73 cm^3^ for AIDR3D (*p =* 0.02) and 0.37 cm^3^ for FBP (*p = 0*.036) reconstructions per 10 % dose increase in patient 1, and by 1.2 cm^3^ (*p =* 0.02) and 0.67 cm^3^ (*p =* 0.058) per 10 % dose increase in patient 2. As a fraction of the reference bone STL model volume, the residual volume for AIDR3D configurations was 2.9 % at 20 % dose and decreased to 1.4 % at 50 % dose in patient 1, and 4.1 % at 20 % dose and decreased to 1.9 % at 50 % dose in patient 2. For FBP configurations, it was 3.3 % at 20 % dose and decreased to 1.0 % at 100 % dose in patient 1, and 5.5 % at 20 % dose and decreased to 1.6 % at 100 % dose in patient 2.

The morphology of the residual volume resembled a thin shell on the osseous surface (Fig. 2c) for all configurations. This shell was the volume wherein the reference STL differed from the alternative configuration-derived STLs. Its average thickness was less than 0.1 mm in all configurations, and was largest at 20 % dose for either AIDR3D or FBP configurations and consistently decreased at increasing simulated radiation dose. For patient 1, it ranged from 0.082 mm (20 % dose) to 0.039 mm (50 % dose) for AIDR3D configurations, and from 0.086 mm (20 % dose) to 0.045 mm (100 % dose) for FBP configurations. For patient 2, it ranged from 0.087 mm (20 % dose) to 0.039 mm (50 % dose) for AIDR3D configurations, and from 0.104 mm (20 % dose) to 0.034 mm (100 % dose) for FBP configurations.

## Discussion

3D printing requires the isolation and modeling of the volume occupied by individual tissues in DICOM images. If 3D printing is implemented in patient care, radiologists will be obligated to interpret those volumes as well as determine and ensure their accuracy. Thus, 3D printing-specific quality metrics will be required to assess 3D medical models, both as a function of the underlying imaging acquisition and pathology involved, as well as of the interpreting radiologist. While phantoms can be used to test the accuracy of 3D printing hardware, to date there are no measures of quality and benchmarks for the accuracy and reproducibility of STL models generated from DICOM images for 3D printing.

**Fig. 7:**
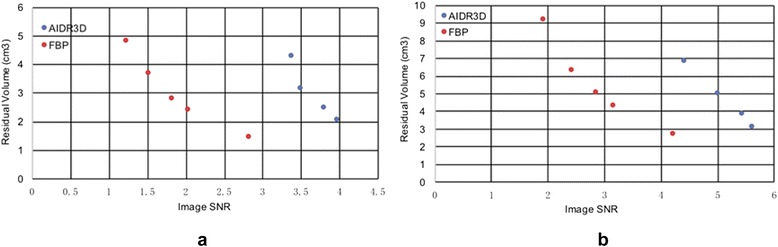
Residual volume as a function of image SNR for AIDR3D and FBP reconstruction in the first (**a**) and second (**b**) patient

This work is based on the authors’ recognition that studies of inter- and intra-observer reproducibility and accuracy will require a conceptually simple metric that correlates to anatomic differences between 3D-printable STL models derived from medical images. The topology of the residual STL volume has two key properties. First, it appears to follow image SNR in an anticipated manner – higher SNR leads to a smaller residual volume (Fig. 7). Second, the residual volume can be used to both visualize and mathematically measure the difference between STL models derived from DICOM image datasets depicting the anatomy.

This is important to help establish the requirements of medical images, in terms of e.g., image SNR, tissue CNR, and image resolution to enable accurate 3D printing. No studies have addressed these requirements to date either in the bone or any other tissue. Guidance regarding such choices is largely based on the experience of the operator [1]. An important application of the residual volume is thus that information extracted from mathematical measures of its shape can be used to guide the selection of imaging protocol and required SNR and CNR to achieve accurate 3D printing. For example, decreasing SNR due to reduced radiation exposure resulted in a “shell” of small additional thickness to the bone STL models studied here. This indicates that the effects of the increased noise for bone 3D printing translate to benign (<0.1 mm in width) uncertainty at the transitions between bone and tissue, rather than bulk anatomic errors.

Similar study designs to that used here can, for example, assess inter-scan variability of 3D printing by applying the residual volume to assess models derived by repeated acquisition of images from a single patient. Similarly the residual volume can be used to assess intra-scan variability with different imaging parameters, e.g., help establish how different slice thicknesses affect anatomic features of interest in the printed model. Such studies will begin to address open issues regarding quality and standardization for 3D printing from DICOM data. Furthermore, strategies based on the residual volume can potentially help evaluate the effect of different segmentation methods or segmentation parameters. Choi et al. used thresholding segmentation cutoffs of 700 HU for the cranio-maxillary complex and 800 HU for the mandible [26] while Salmi et al. used a 500 HU cutoff for the entire skull and mandible [27], although both used CT datasets acquired at 120 kVp. The residual volume can be used to compare models derived from threshold ranges other than the widely-accepted software-default HU range for bone used here. Of note, the topology of the residual volume can be readily interrogated at pre-specified locations of particular clinical interest, such as along the axis of the mandibular body length used in orthognathic and reconstructive surgeries [26] or at the anatomic limits of a tumor [6], a region where manual segmentation, including decisions best suited for the radiologist, is important for diagnosis and surgical planning.

Just as the initial volume rendering of bony structures portrayed on a 2D monitor [28] inspired the field of 3D visualization and ushered the development of the “3D lab” in radiology, “3D printing” of radiology images has roots in skeletal radiology, including a more than 20 year history of in cranio-maxillofacial reconstruction [2, 29]. Our data also concentrates on cranio-maxillofacial bone. CT bone segmentation is relatively straightforward because of its high attenuation and signal. Its “hard” separation form adjacent tissues aids 3D printing-specific STL refinements such as smoothing that are often employed to convert medical DICOM images into reasonable STL files [1]. Deviations from anatomy and pathology that can alter the anatomy portrayed in the 3D printed model can arise not only from such computer-aided design refinements, but from numerous factors including the quality of the source images, image post-processing techniques including segmentation technique, and STL generation algorithms that determine the set of triangles that will be used to represent the surface encompassing the segmentation. A large source of potential error also lies in the additive manufacturing process itself and its limitations, including the particular 3D printing modality and the selection of printing materials [1].

The residual volume is designed as a metric that enables either the ensemble or individual assessment of each acquisition, post-processing, and STL generation factor involved in 3D printing, so that individual models or practices to generating them can be assessed for e.g., reproducibility and accuracy before they are implemented to clinical practice. This expands the scope of prior accuracy studies, for example those that have concentrated on comparing a final printed model with the posthumous anatomy used to provide source DICOM images [30, 31], or those that have compared physical measurements to those made with 3D post-processing software in the source images [6, 26, 27].

This work applied the residual volume to assess radiation dose reduction [32] for 3D printing. Automated CT tube current and tube potential selection combined with iterative reconstruction methods optimize the tradeoffs between radiation exposure and SNR [33], and have led to customized patient-centric imaging strategies. The residual volume demonstrates that a large reduction in radiation dose, using just 20 % of that used for surgical planning, has a very minor effect in the generation of 3D printable models of maxillofacial bone. The average difference of less than 0.1 mm was smaller than the spatial resolution of the CT hardware and of the typical dimensional error (0.5–0.9 mm) of the 3D printing process itself [26, 27, 31]. Our data thus mathematically confirms that current low-dose CT protocols meet the technical needs for 3D printing of bone.

While we propose that the residual volume can be translated to all applications of 3D printing from DICOM images, we expect that results for soft tissue models will vary in comparison with those from bone because of the different SNR and CNR properties. Our preliminary work suggests that reduced arterial contrast opacification in contrast-enhanced CT angiography for example leads to large portions of vessels such as the aorta being excluded from semi-automated segmentation, leading to much larger residual volumes that we report for bone. More work is needed to assess the use of the residual volume in soft tissues and the contrast-enhanced blood pool and to similarly establish the impact of varying the image acquisition and segmentation parameters.

### Limitations

The maxillofacial bone STL experiments were enabled by specialized patients undergoing full-face transplantation at our institution. Raw CT data was archived, enabling the generation of a spectrum of simulated lower dose images. Although reduction of dose to the 20 % level may allow bone 3D printing, soft tissue assessment is limited by high image noise. Furthermore, although the simulated dose reduction has been validated [21], consecutive CT acquisitions at varying doses would be required to confirm our results in practice. We acknowledge the small sample size, although results were extremely consistent across both patients and all bone structures and they are thus likely generalizable to cranio-maxillofacial bone 3D printing. Also, for maxillofacial applications, implantable devices are of increasing importance, and this work is limited in scope to surgical planning only.

## Conclusions

This study introduces the residual volume, a simple topological difference metric of STL models generated from a tissue depicted in DICOM images. The residual volume is designed to enable the ensemble or individual assessment of each acquisition, post-processing, and STL generation factor involved in 3D printing, so that individual models or practices to generating them can be assessed for e.g., reproducibility and accuracy before they are implemented to clinical practice. The topology of the STL residual volume has two key properties; first, it appears to follow image SNR in an anticipated manner – higher SNR leads to a smaller residual volume, and second, the residual volume can be used to both visualize and mathematically measure the difference between STL models derived from DICOM image datasets depicting the anatomy. Information extracted from mathematical measures of the shape of the residual volume can be used to guide the selection of imaging protocol in terms of required resolution, SNR and CNR toward ensuring accurate 3D printing. Finally, application of the residual volume in musculoskeletal 3D printing supports that lower dose images of bone can be accurately 3D printed. A reduction of the tube current by up to 80 % in conjunction with iterative reconstruction results in bone STL models that have a small difference in bone volume of less than 4 %, concentrated in a shell surrounding it that has less than 0.1 mm thickness.
